# PASTRY: achieving balanced power for detecting risk and protective minor alleles in meta-analysis of association studies with overlapping subjects

**DOI:** 10.1186/s12859-023-05627-z

**Published:** 2024-01-12

**Authors:** Emma E. Kim, Chloe Soohyun Jang, Hakin Kim, Buhm Han

**Affiliations:** 1 Department of Chemistry, Seoul National University, Seoul, 03080 Korea; 2 Department of Biomedical Sciences, Seoul National University College of Medicine, Seoul, 03080 Korea; 3 Interdisciplinary Program for Bioengineering, Seoul National University, Seoul, 03080 Korea

**Keywords:** Methods, Meta-analysis, GWAS, Overlapping subjects, Correlation

## Abstract

**Background:**

Meta-analysis is a statistical method that combines the results of multiple studies to increase statistical power. When multiple studies participating in a meta-analysis utilize the same public dataset as controls, the summary statistics from these studies become correlated. To solve this challenge, Lin and Sullivan proposed a method to provide an optimal test statistic adjusted for the correlation. This method quickly became the standard practice. However, we identified an unexpected power asymmetry phenomenon in this standard framework. This can lead to unbalanced power for detecting protective minor alleles and risk minor alleles.

**Results:**

We found that the power asymmetry of the current framework is mainly due to the errors in approximating the correlation term. We then developed a meta-analysis method based on an accurate correlation estimator, called PASTRY (A method to avoid Power ASymmeTRY). PASTRY outperformed the standard method on both simulated and real datasets in terms of the power symmetry.

**Conclusions:**

Our findings suggest that PASTRY can help to alleviate the power asymmetry problem. PASTRY is available at https://github.com/hanlab-SNU/PASTRY.

**Supplementary Information:**

The online version contains supplementary material available at 10.1186/s12859-023-05627-z.

## Introduction

Genome-wide association studies (GWAS) have identified numerous variants associated with traits. However, early studies suffered from the limitation of using small sample sizes, which made them underpowered to detect variants with small effect sizes. Larger sample sizes are required to address this challenge, but increasing sample size can often be difficult for a single researcher. The implementation of meta-analysis, a statistical technique that combines multiple GWAS summary statistics, has proven to be instrumental in increasing the sample size and enhancing the statistical power to detect more associated variants. Specifically, the fixed effects model is widely acknowledged as the prevailing methodology for conducting the meta-analysis of multiple studies [1–3].

A common requirement of meta-analysis is that the participating samples have to be independent among studies [2]. When multiple studies in a meta-analysis have overlapping controls, the resulting summary statistics become correlated, leading to an increased risk of false positives. However, this independency requirement is often violated in GWAS meta-analysis because studies often utilize the same public datasets as additional controls [4–14].

Fortunately, recent advances in meta-analysis methods have addressed this issue by explicitly accounting for the correlations arising from shared subjects. Lin and Sullivan introduced a correlation estimator coupled with an optimal test statistic to account for the correlations [15]. Their method has demonstrated comparable power to the splitting approach, which refers to an imaginary method that divides shared individuals into the respective studies prior to the meta-analysis. After Lin and Sullivan’s method was proposed, several additional methods were also developed, but they were based on a similar correlation estimator [16–18]. Thus, the correlation estimator suggested by Lin and Sullivan and their associated method have become the standard practice to deal with overlapping samples in GWAS meta-analysis.

In this paper, we report a phenomenon that the use of this standard framework suggested by Lin and Sullivan [15] can lead to unbalanced power for detecting protective minor alleles (Relative risk; RR < 1) and risk minor alleles (RR > 1). We observed that when the controls were shared among studies in meta-analysis, the power for detecting protective minor alleles became severely lower than the power for detecting risk minor alleles. In our simulation of five-study meta-analysis for testing a SNP with a minor allele frequency (MAF) of 0.1, when the minor allele’s effect was risk (RR = 1.30), the standard framework showed 80.4% power. However, when we reversed the effect direction (RR = 1/1.30), the power decreased to 56.0%. As MAF decreased, the degree of power asymmetry worsened. In contrast, the splitting approach based on genotype data did not show this phenomenon and consistently achieved 69.5% power for both situations.

Having an unbalanced power for detecting risk and protective minor alleles can adversely impact the interpretability of downstream analyses. The presence of a higher number of risk minor alleles, as demonstrated by Chan et al. in 2014 [19], can provide evidence for polygenic inheritance in complex diseases. Furthermore, the imbalance between risk and protective minor alleles can offer insights for population genetic analyses, including analyses of selective pressure in relation to a specific disease [20]. These analyses are typically based on the assumption that commonly used two-sided tests have equal power for detecting risk and protective minor alleles. Therefore, if the association results were generated by a severely unbalanced test, the results can mislead interpretation.

We investigated why the power asymmetry phenomenon occurs. We found that the standard correlation estimator of the current method was approximated under the null hypothesis of no effect, and this approximation led to an imperfect estimate. It turns out that the true correlation is largely dependent on the MAF and effect size under the alternative hypothesis, and simply ignoring them could lead to substantially unbalanced power. To overcome this problem, we developed a method called PASTRY (A method to avoid Power ASymmeTRY). Our method is built upon an accurate correlation estimator that accounts for both MAF and effect size. By simulations and real data analyses, we show that PASTRY substantially reduces the power asymmetry phenomenon in meta-analysis with overlapping samples.

## Methods

### Case–control GWAS model

Case–control GWAS studies use a logistic regression model for finding disease-associated SNPs, where the binary trait represents individuals as either "cases" (disease) or "controls" (no disease). The simple logistic regression model used for a binary outcome is as follows:

1 $$logit\left(\varvec{Y}\right)=ln\left(\varvec{odds}\right)= \varvec{\alpha} +\varvec{\beta} \varvec{X} +\varvec{\epsilon}$$

2 $$\mathrm{Pr }\left(Y=1|X\right)= \frac{{e}^{\hat{\alpha} +\hat{\beta }X}}{1+{e}^{\hat{\alpha} +\hat{\beta} X}}$$

where $p$ denotes the probability that the (case) event will occur, $Y\in \{\mathrm{0,1}\}$ is the disease status, $X\in \{\mathrm{0,1}\}$ is the explanatory variable (i.e., genotype dosage of one SNP), and $\alpha$ and $\beta$ are the intercept and regression parameter.

Suppose there are $M$ case–control GWAS studies and we are interested in combining these summary-level results into a single estimate. In general, the random effects model is often favored over the fixed effects model to account for heterogeneity. However, we see a different trend in GWAS meta-analyses where the fixed effects model tends to be more commonly used [2, 21–24]. Before describing our PASTRY model, we describe the fixed effects model for the GWAS meta-analysis and its extension, Lin and Sullivan method (LS).

### Fixed effects (FE) model

The fixed effects model assumes that the effect sizes are the same across all the studies. A common method for this model is the Inverse Variance-Weighted (IVW) average method, which combines the effect sizes by weighting them by the inverse of their variance. The IVW estimator can be represented as follows:

3 $${\hat{\beta }_{IVW}}= \frac{\sum {W}_{i}\hat{{\beta }_{i}}}{\sqrt{\sum {W}_{i}}},$$

where $i$ is the index for the study ($i=\mathrm{1,2},\dots ,M$), $\hat{{\beta }_{i}}$ is the effect size of the study $i$, and the weights are defined as

4 $${W}_{i}= \frac{1}{\hat{{\sigma }_{i}^{2}}}$$

where $\hat{{\sigma }_{i}^{2}}$ is the variance of $\hat{{\beta }_{i}}$. The variance of this estimator turns out to be

5 $$Var\left(\hat{{\beta}}_{IVW}\right)=\frac{1}{\sum {W}_{i}} .$$

### Lin and Sullivan’s (LS) method

Lin and Sullivan developed a fixed-effects model method that can account for the correlation introduced by overlapping samples in meta-analyses. Here, we will refer to this method as LS for abbreviation. First, Lin and Sullivan analytically derived the approximated correlation formula [15]. Then, the final meta-analysis statistic is obtained after accounting for the cross-study correlations. Suppose that we have $M$ studies $(1,..k,l,..M; l\ne k)$ with observed effect sizes of $\widehat{{\varvec{\beta}}}=(\widehat{{\beta }_{1}},\dots ,\widehat{{\beta }_{M}})$ in the meta-analysis. Lin and Sullivan showed that the correlation between statistics of study $k$ and $l$ is approximately a function of the sample sizes:

6 $${r}_{kl}\approx \frac{{{n}_{kl-}\sqrt{\frac{{{n}_{k+}n}_{l+}}{{{n}_{k-}n}_{l-}}}} + n_{kl+}\sqrt{\frac{{{n}_{k-}n}_{l-}}{{{n}_{k+}n}_{l+}}}}{\sqrt{{n}_{k}{n}_{l}}}$$

${n}_{k}$ and ${n}_{l}$ denote the total number of samples in study $k$ and $l$, and the subscript + and – define case and control specific sample sizes. ${n}_{ij+}$ and ${n}_{ij-}$ denote the number of overlapping case and control subjects between study $i$ and $j$, respectively. If we wish to combine $M$ studies with overlapping samples, we can build a $M\times M$ correlation matrix ${\text{C}}$, where element $[k,l]$ is the correlation between studies $k$ and $l$, ${r}_{kl}$.

7 $${\text{C}}={\left[{r}_{kl}\right]}_{M\times M}$$

The $M\times M$ variance–covariance matrix $\Omega$ can be obtained using the correlation matrix above and the standard deviations of studies. Then, the meta-analyzed effect size, ${\hat{\beta }_{LS}}$, and the variance $Var(\hat{{\beta}}_{LS})$ can be calculated as

8 $${\hat{\beta}}_{LS}=\frac{{{\varvec{e}}}^{T}{{\varvec{\Omega}}}^{-1}\widehat{{\varvec{\beta}}}}{{{\varvec{e}}}^{T}{{\varvec{\Omega}}}^{-1}{\varvec{e}}} ,$$

9 $$Var\left(\hat{{\beta}}_{LS}\right)=\frac{1}{{{\varvec{e}}}^{T}{{\varvec{\Omega}}}^{-1}{\varvec{e}}} ,$$

where ${\varvec{e}}$ is the length-$M$ vector of ones.

### PASTRY method

#### Effect size and variance of PASTRY

The meta-analysis effect size and the variance of our new method PASTRY have very similar forms to the LS method, as follows.

10 $${\hat{\beta}}_{PASTRY}=\frac{{{\varvec{e}}}^{T}{{\varvec{\Omega}}}_{PASTRY}^{-1}\widehat{{\varvec{\beta}}} }{{{\varvec{e}}}^{T}{{\varvec{\Omega}}}_{PASTRY}^{-1}{\varvec{e}}} ,$$

11 $$Var\left(\hat{{\beta}}_{PASTRY}\right)=\frac{1}{{{\varvec{e}}}^{T}{{\varvec{\Omega}}}_{PASTRY}^{-1}{\varvec{e}}} .$$

The difference is that we use a different variance–covariance matrix, namely ${\Omega }_{{\text{PASTRY}}}$ [15]. This matrix has the following form:

12 $$\Omega_{{PASTRY_{k,l} }} = I_{PASTRY_k}^{ - 1} Cov_{PASTRY} \left( {U_{k} ,U_{l} } \right) I_{PASTRY_l}^{ - 1}$$

where $\Omega_{{PASTRY_{k,l} }}$ represents the variance–covariance between two studies ($k$ and $l$), as well as the classical robust sandwich variance estimator [25].

${{I}_{PASTRY_k}}$ denotes the information matrix in study $k$, $Cov_{PASTRY}\left({U}_{k},{U}_{l}\right)$ is the covariance between the two score functions of studies $k$ and $l$. In Appendix, we elucidated the procedures for deriving the detailed formula.

### Splitting approach

The splitting approach is the most naïve and simple method to deal with overlapping samples. This method splits the overlapping samples into individual studies before meta-analysis, so that all samples can be non-overlapping. Although this method obviously solves the overlapping sample problem, this method is impractical in many situations because individual studies are already performed and cannot be modified. Although impractical, for performance comparison, we included this method in Results.

### Power simulation

We conducted power simulations to compare methods. We assumed that we combine $K$ studies. In each simulation, we assumed each study had $n$ samples consisting of ${n}_{+}$ cases and ${n}_{-}$ controls and all controls were shared among studies. We assumed a variant with a MAF of $p$. Assuming a very low prevalence, the expected case MAF becomes ${p}^{+}= \gamma p/((\gamma -1)p+1)$ and the expected control MAF becomes ${p}^{-}\approx p$, where $\gamma$ refers to relative risk. We randomly sampled the number of minor alleles for cases and controls using the binomial distribution.

For the splitting approach, we ensured that the sum of the minor allele counts of the overlapping samples were the same before and after splitting. We conducted simulations under different scenarios, varying the number of studies (from 2 to 10), MAF (from 0.1 to 0.5), relative risk (risk: 1.05, 1.10, 1.15, 1.20, 1.25, 1.30; protective: 1/1.30, 1/1.25, 1/1.20, 1/1.15, 1/1.10, 1/1.05). We iterated each simulation 100 K times to assess the power of the methods.

### Real data analysis

#### UK biobank diabetes mellitus data

We used the UK Biobank data project (www.ukbiobank.ac.uk) for real data analysis. The data contains 488,377 individuals and 784,256 autosomal genotyped genetic markers. We used a diabetes mellitus phenotype (Illness code E10-14 Diabetes mellitus in field 41,202 and 41,204) to evaluate the PASTRY, LS method, and the splitting approach.

First, we performed a GWAS analysis using logistic regression model implemented in PLINK. There are 368,329 people in control set and 30,220 in case set. We identified 468 statistically significant loci from the GWAS results and focused on the candidates (Additional file 1: Table S1).

Second, we randomly split the control and case samples into 5 groups. We treated each group as an independent study and conducted a GWAS analysis on each study. In the splitting approach, we used 73,700 and 6040 individuals for control and case in each study. In contrast, in the PASTRY and LS methods, we conducted a meta-analysis of studies with shared control design where all controls are shared. We used 368,329 shared controls and 6044 cases for each set in the shared design. We applied three meta-analysis frameworks: the PASTRY method, LS method, and the splitting approach.

Third, we calculated and visualized the ratio of *p*-values of the PASTRY method (and LS method) over the *p*-value of splitting approach for the 768 loci for six categorized ranges of odds ratios (ORs): − 1.20, 1/1.20–1/1.10, 1/1.10–1.00, 1.00–1.10, 1.10–1.20, 1.20–.

#### WTCCC data analysis

We also used data from Wellcome Trust Case Control Consortium 1 (WTCCC, 2007) for real data analysis [4]. The data consist of ~ 2000 case samples for each of seven diseases, and one shared ~ 3000 control samples. We only used data for type 1 diabetes (T1D), rheumatoid arthritis (RA), and Crohn's disease (CD). We followed the full overlap design from the FOLD study [26], which performed a GWAS by fitting a logistic regression model to the genotype data for each of the three diseases. After quality control, 1748 CD samples, 1860 RA samples, and 1963 T1D samples were left. We extracted eight significant loci related to the three autoimmune diseases (Additional file 1: Table S2). Two of these loci were identified in the WTCCC GWAS, and the other six were identified in ImmunoBase (http://www.immunobase.org). After applying three methods, we calculated the *p*-value ratios of PASTRY (and LS method) over the *p*-value of splitting approach for eight loci.

## Results

### Unexpected power asymmetry of the standard framework

We identified an asymmetry in the power of Lin and Sullivan's (LS) method, which is the most widely used framework for addressing sample overlap in meta-analysis. LS method can be considered the standard framework, since this was the first method that derived the correlation estimator, and the similar estimator was adapted by subsequent methods. We compared the power of LS method to the splitting approach, which splits overlapping samples into separate studies. Splitting method is undoubtedly the simplest solution to deal with sample overlap, but it is not applicable in practice because only summary statistics, not the genotype data, are available for meta-analysis in most situations.

We used the following simulation scheme. We generated a meta-analysis of five studies ($K=5$) where each study had 3,000 cases and 3,000 controls (${N}_{+}={N}_{-}=\mathrm{3,000}$) for all studies, assuming that all controls were shared among studies. We considered two scenarios. First, we varied the minor allele frequency (MAF) from 0.1 to 0.5. In this scenario, we assumed a relative risk (RR) of 1.30 for a risk allele and 1/1.30 for a protective allele. Second, we varied RR. We simulated different RRs from 1.05 to 1.30 for the risk allele, and from 1/1.05 to 1/1.30 for the protective allele. In this scenario, we fixed MAF as 0.1. We replicated each simulation setting 100 K times to estimate the power for LS method and the splitting approach. Here, we adjusted the significance threshold to maintain the overall power of the splitting approach at approximately 70%.

Before evaluating the power of PASTRY, we assessed the false positive rate (Additional file 1: Figure S1) at various minor allele frequencies (MAFs). Our false-positive rate simulations demonstrated that our method consistently maintains a well-controlled Type I error rate under a range of diverse MAFs.

**Fig. 1:**
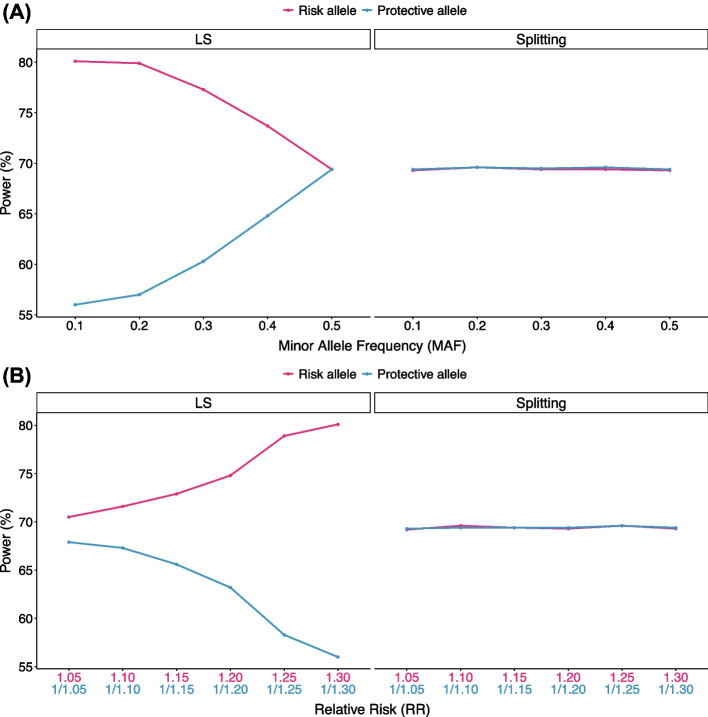
Powers of LS method and splitting approach with different minor allele frequencies (MAF) and relative risks (RRs). **A** We assessed the power of the LS method and the splitting approach as we varied MAF from 0.1 to 0.5. In this case, we assumed risk minor alleles (RR = 1.30; pink) and protective minor alleles (RR = 1/1.30; skyblue). **B** Also, we assessed the power of the LS method and the splitting approach as we varied the RRs from 1.05 to 1.30 for risk minor alleles (pink) and 1/1.05 to 1/1.30 for protective minor alleles (skyblue). In this case, we assumed MAF of 0.1

Figure 1A illustrates the first scenario where we varied MAF. In the case where the allele was protective (RR = 1/1.30), the power of the LS method was lower than that of the splitting approach. The power difference was the greatest at the small MAF of 0.1. At this MAF, the power of LS was 56%, while that of splitting was 69%. In the case where the allele was risk (RR = 1.30), the power of the LS method was higher than that of the splitting approach. Again, the power difference was the greatest at the small MAF of 0.1. At this MAF, the power of LS was 80%, while that of splitting was 69%. These results implied that at MAF of 0.1, depending on the direction of effects, the power of LS method can drastically vary between 56 and 80%. When we examined the EUR dataset of 1000 Genomes phase3 data on GRCh38, 76,014,324 out of 84,805,772 SNPs (89.63%) had MAF < 0.1. Thus, if one uses LS method for these SNPs, the power for detecting risk minor alleles and the power for detecting protective minor alleles will be dramatically different.

Figure 1B illustrates the second scenario where we varied RRs. As the absolute value of RR increased, the difference in power between the LS method and the splitting approach became more pronounced. At the largest RR we simulated (RR: 1.3 or 1/1.3), the power of LS varied between 56 and 80% (This was an equivalent situation that we observed in Fig. 1A). Thus, overall, our results showed that the power asymmetry of LS method exists, and the degree of asymmetry was exacerbated as the MAF decreased and as the magnitude of RR increased.

### Comparison of correlation estimators

We investigated on why the power asymmetry occurs in the standard framework and found that the errors in the approximated correlation estimator can be the cause. We considered the simulation in Fig. 1A and B, assuming five studies with all controls are overlapped. In this situation, the correlation estimator of LS was turned out to be exactly 0.5. When one applies LS method, this constant estimator is used for all SNPs regardless of the minor allele's RR or the MAF, because LS's formula solely depends on sample sizes. However, when we apply our method PASTRY, which calculates more accurate correlation taking into account both MAF and effect sizes, different estimate of correlation is used for each SNP.

Tables 1 and 2 show the correlation estimates obtained by PASTRY and LS. At the RR of 1.3 (or 1/1.3), the correlation estimator of PASTRY was 0.573 for risk minor alleles (RR: 1.3) and 0.464 for protective minor alleles (RR: 1/1.3). Thus, the difference of correlation between the two alleles was 0.109. One may argue that the difference of correlation estimator of PASTRY (0.573 or 0.464) compared to LS (0.5) is overly small to make any meaningful difference in power. However, a small difference in correlation estimator can indeed change the results, because the small errors can accumulate from the whole correlation matrix, as we show below.

**Table 1:** Comparison of the correlation from the PASTRY and LS with various minor allele frequencies

Minor allele frequency (MAF)	LS correlation	PASTRY correlation of Risk minor allele (RR = 1.30)	PASTRY correlation of protective minor allele (RR = 1/1.30)
0.1	0.5	0.572	0.464
0.2		0.539	0.454
0.3		0.522	0.467
0.4		0.509	0.484
0.5		0.496	0.495

**Table 2:** Comparison of the correlation from the PASTRY and LS with various relative risk, assuming MAF of 0.1

Allele type	Relative risks (RRs)	LS correlation	PASTRY correlation
Risk minor allele	1.05	0.5	0.521
	1.10		0.535
	1.15		0.545
	1.20		0.523
	1.25		0.534
	1.30		0.572
Protective minor allele	1/1.05		0.500
	1/1.10		0.466
	1/1.15		0.467
	1/1.20		0.442
	1/1.25		0.465
	1/1.30		0.464

### Cumulative effect of inaccuracy in correlation

We investigated the cumulative effect of inaccurate correlation on the final meta-analysis statistics. We used a similar simulation setting as above, with a fixed sample size for each study (${N}_{+}$ = 3000 and ${N}_{-}$ = 3000) with fully overlapped controls. We assumed a MAF of 0.1, and a relative risk (RR) of 1.30 for risk alleles and 1/1.30 for protective alleles.

For simplicity, we assumed the use of LS. Thus, the correlation estimator was fixed as 0.5 by the LS formula. We then added an error term $e$ to the correlation. We assumed a diverse range of error ($e=\Delta r$) from 0 to 0.1. Finally, we varied the number of studies ($K$) from 2 to 10, and measured how the power of LS changes depending on $e$ and $K$.

**Fig. 2:**
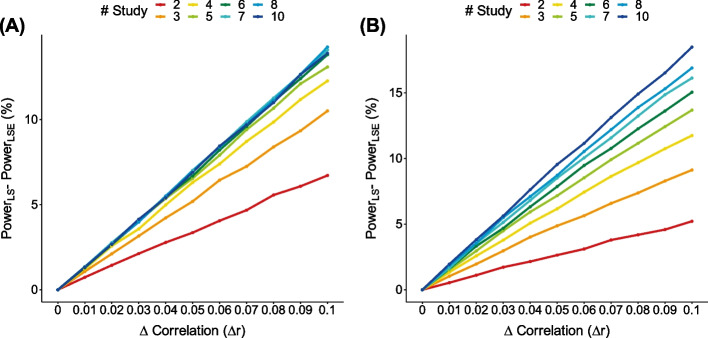
Power difference between the original LS method and the LS method with correlation errors (LSE) for **A** risk (left) and **B** protective (right) alleles, respectively. The correlation errors $(\Delta r)$ were varied from 0.0 to 0.1, and the number of studies was varied from 2 to 10 for both situations

Figure 2A and B show the power difference between the original LS method and the LS method with correlation errors (LSE in short). As expected, the power difference became greater as the error ($e$) increased. Notably, we observed that the power difference depended on the number of studies ($K$). For example, for protective alleles, when $e=0.1$, the power difference was 18% with the number of studies of 10, while it was only 5% with the number of studies of 2. This result demonstrates that a small error in correlation ($e$) can have dramatic impact on the final power if $K$ is large, because the impact of errors can accumulate over the $K\times K$ correlation matrix.

### PASTRY achieves similar power to the splitting approach

**Fig. 3:**
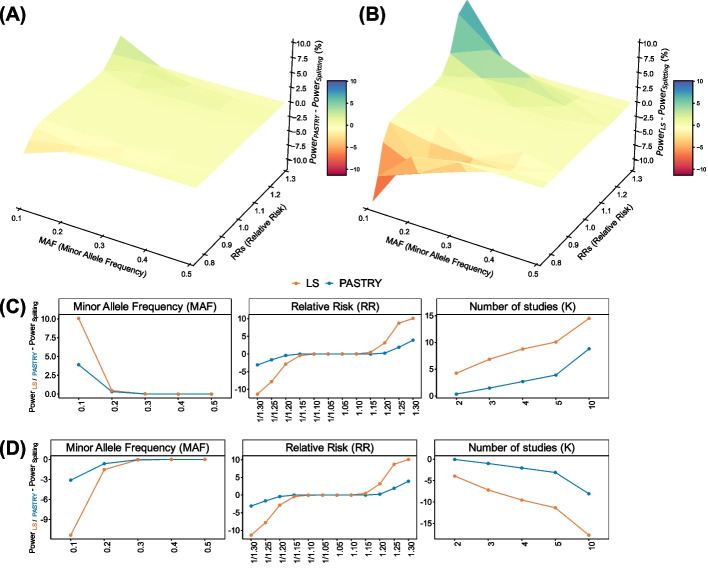
Power difference of PASTRY and LS methods over splitting approach in various settings. **A, B** Three-dimensional surface plots of the power difference between **A** PASTRY and **B** LS methods over splitting approach for various MAFs and RRs. **C, D** Line plots comparing the power difference between PASTRY and LS methods over splitting approach for **C** risk minor alleles and **D** protective minor alleles in various settings

We evaluated the power of PASTRY in a variety of situations, while varying minor allele frequency (MAF), relative risk (RR), and the number of studies ($K$). We assumed $K$ studies, each with 3000 samples for both cases and controls with fully overlapped controls. Specifically, we considered four scenarios. First, we varied both MAF (from 0.1 to 0.5) and RRs (from 1/1.3 to 1.3) while keeping the number of studies to 5 ($K$ = 5). This gave us power estimates of methods over the two-dimensional space of parameters. Second, we only varied MAF (from 0.1 to 0.5) while keeping the number of studies to 5 ($K$ = 5) and keeping RR to 1.30 (or 1/1.30). Third, we only varied RR from 1.05 to 1.30 (or 1/1.05 to 1.30) while keeping MAF to 0.1 and the number of studies to 5 ($K$ = 5). Fourth, we only varied the number of studies ($K$) from 2 to 10 while keeping MAF to 0.1 and RR to 1.30 (or 1/1.30). For each scenario, we calculated the power difference of PASTRY compared to the splitting approach (Fig. 3). Additionally, we also calculated the power difference of LS compared to the splitting approach.

Before evaluating the power of PASTRY, we assessed the false positive rate (Additional file 1: Table S3) at various minor allele frequencies (MAFs) and the number of studies ($K$). Our false-positive rate simulations demonstrated that our method consistently maintains a well-controlled Type I error rate under a range of diverse conditions.

Figure 3A and B illustrate the first scenario where we varied both MAF and RR. As expected, PASTRY (3A) generally achieved similar power to splitting, while LS (3B) deviated from splitting. For example, when MAF was 0.1 and RR was 1.3 (or 1/1.3), the power difference of PASTRY was only 3.90 for risk minor allele and −3.10% for protective minor allele. In contrast, the LS method showed power difference of 10.07% for risk minor allele and −11.34% for protective minor allele. The power of both methods tended to deviate from splitting as MAF decreased and RR moved further away from 1. However, the deviation was much greater for LS than PASTRY.

Figure 3C and D show the second, third, and fourth scenarios for risk and protective alleles, respectively. In the second scenario, the power difference between PASTRY and LS was the largest when the MAF value was 0.1. As the MAF value increases, LS and PASTRY showed similar power. In the third scenario, the power difference between PASTRY and LS was the largest when the effect size was greater (RR value of 1.30 or 1/1.30). In the last scenario, the power difference between PASTRY and LS was the largest when the number of studies was greater ($K=10$). In addition, we compared the results of PASTRY method and LS method in a wider range of settings (Additional file 1: Table S3). In sum, LS showed considerable power difference from splitting, of which the magnitude of difference was increased as MAF becomes lower, effect size becomes larger, and the number of studies becomes larger. Although PASTRY also showed power difference from splitting, the magnitude of difference was much smaller than that of LS. These results suggest that PASTRY can alleviate the power asymmetry problem that the current standard framework (LS) has.

### Application to diabetes mellitus dataset from UK Biobank data

We evaluated the performance of PASTRY method using the diabetes mellitus data from the UK Biobank dataset. This dataset had 768 significant loci, and we only focused on these loci (Additional file 1: Table S1). We split the cases into five groups to make five studies, which were designed to share the whole controls (See Methods). Using meta-analysis methods (PASTRY and LS), we obtained the *p*-values of the significantly associated SNPs. We then compared the *p*-values of PASTRY and LS to the splitting approach by calculating the ratio of *p*-values. We evaluated the *p*-value ratios per each bin of effect size (OR).

**Fig. 4:**
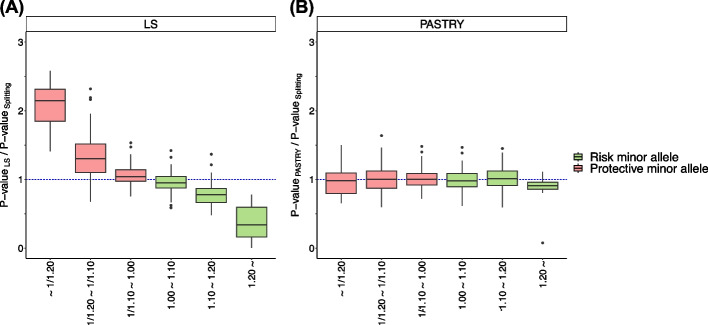
Comparison of *p*-value ratios of **A** PASTRY method and **B** LS method over the splitting approach for diabetes mellitus dataset from UK Biobank data. The x-axis shows the odds ratios (ORs) of the significant loci, and the y-axis shows the *p*-value ratios. The green boxes show the *p*-value ratios for the risk minor alleles, and the pink boxes show the *p*-value ratios for the protective minor alleles. The protective minor alleles and risk minor alleles were divided into three ranges each (protective: − 1/1.20, 1/1.20–1/1.0,1/1.10–1.00 and risk: 1.00–1.10, 1.10–1.20, 1.20− ). The blue dashed line represents y = 1, where the two methods’ *p*-values are the same

Figure 4 shows the ratios of *p*-values for risk minor alleles (OR > 1) and protective minor alleles (OR < 1). We divided each into three ranges (protective: −1/1.20, 1/1.20–1/1.0,1/1.10–1.00 and risk: 1.00–1.10, 1.10–1.20, 1.20–). A *p*-value ratio closer to 1 indicates better performance of the corresponding method, because it means that the power is closer to the splitting method and therefore there is less degree of power asymmetry. Consistent with the previous simulation results, LS method (4A) tended to give smaller *p*-values for risk minor alleles and larger *p*-values for protective minor alleles. The absolute value of the ratio increased as the OR moves away from 1. In contrast, PASTRY method (4B) generally maintained a median close to 1. For example, for SNPs with OR value of 1/1.20 or smaller, the median value of the *p*-value ratio of LS was 2.189, while the median of PASTRY was 0.985. For SNPs with OR value of 1.20 or greater, the median value of the *p*-value ratio of LS was 0.339, while the median value of PASTRY was 0.912. Thus, PASTRY method outperformed LS method in this real data analysis, in terms of the power symmetry.

### Application to WTCCC data

Lastly, we performed a similar real data analysis using the Wellcome Trust Case Control Consortium (WTCCC) data. We conducted the same cross-disease analysis as Kim et al. who meta-analyzed three autoimmune diseases: Crohn's disease (CD), rheumatoid arthritis (RA), and type 1 diabetes (T1D), treating them as three studies (see Methods for details) [26].

To assess the Type I error rate and the presence of truly effect SNPs, we first generated quantile–quantile (QQ) plots for the meta-analysis of the three different models for all SNPs. Additional file 1: Figure S2 shows the QQ plots for the meta-analysis of three different models for all SNPs. These plots show that the most of points are generally close to the diagonal line, which indicates that our method is maintaining the correct Type I error rate. However, there are some strong deviations from the diagonal line with extremely low *p*-values, which suggests that there may be SNPs with true effects on the phenotype.

For the eight candidate loci defined by Kim et al., we calculated the *p*-values of splitting method, LS, and PASTRY. For LS and PASTRY, we assumed the full overlap of controls. Additional file 1: Table 2 shows that the ratios of the *p*-values for the PASTRY method over the splitting approach were all closer to 1 compared to the ratios for the LS method. Thus, consistent to previous analyses, this analysis also showed that PASTRY achieved similar *p*-values as splitting and has less degree of power asymmetry problem.

## Discussion and conclusions

In this paper, we proposed a method that uses an accurate correlation estimator, called PASTRY (A method to avoid Power ASymmeTRY). We identified a phenomenon that the widely-used method (LS) can lead to asymmetry in power for detecting protective and risk minor alleles. We investigated this problem and found that this phenomenon was mainly due to incorrect correlation approximation. We then developed PASTRY, which uses more accurate correlation estimator that accounts for both MAF and effect size. Using simulations, we showed that power asymmetry can be alleviated by using our proposed method PASTRY. Real data analysis using the UK Biobank and WTCCC data also produces concordant results.

However, there are some limitations to consider regarding our approach. Our study modeled genotype as 0 and 1, assuming the multiplicative model to be true (additive model under the log scale). Under the multiplicative model, the allelic model (0/1) and the genotypic model (0/1/2) give the same asymptotic power under the HWE [27]. However, when the model deviates from the multiplicative model, genotypic modeling will be more powerful. Similar to the Lin-Sullivan study from which we derive our method as an extension, we employed the allelic model in our study. However, it will be possible to extend our method to incorporate the genotypic model in the future.

Another limitation of our approach is that PASTRY requires separate MAFs for cases and controls of each SNP. In real-world applications, it might not always be possible to access distinct MAFs for cases and controls directly, especially when dealing with summary statistics data sets. However, it is possible to recalculate the case and control MAF using the population MAF and prevalence. Since the population MAF is available through public data such as 1000 Genomes project and the prevalence is available for many diseases for different ancestries, we expect that this additional information will be obtainable.

A final limitation of our approach is that it can only be applied for genotype-based case–control studies. The challenge of overlapping subjects is indeed a significant issue that also arises in meta-analyses of clinical trials or cohort studies. At present, PASTRY is primarily designed for genotype-based case–control studies due to its specific methodology and underlying assumptions. However, extending PASTRY to non-GWAS studies will be a fascinating avenue worth exploring in the future studies.

Our method PASTRY method extended LS method, but they differ in their input statistics. The LS method requires the effect size and standard errors from each study, as well as the number of subjects and overlapping subjects. The PASTRY method, in addition to these inputs, also requires separate MAFs for cases and controls for each SNP. This additional requirement is necessary for PASTRY to account for potential differences in allele frequencies between cases and controls.

There are several specific conditions that PASTRY and LS will give the same output. As the correlation difference between PASTRY and LS is a function of effect size (beta), when the effect size estimate used by PASTRY is zero, the two statistics will be equal. Another condition is when there are no overlapping subjects. Under this condition, no correlation exists, and therefore the two methods will give an identical result.

To our knowledge, our study is the first study that reported the power asymmetry phenomenon of the standard framework. Moreover, our study is the first to discover the primary cause (error in correlation estimator) and to provide a possible solution. However, the limitation of our approach is that although the power asymmetry was considerably alleviated by our method, the correction was not perfect. Even with our method, there was a small amount of difference in power compared to splitting. This could be because our PASTRY estimator is still not perfect, or because there can be other causes.

A philosophical question might be whether we really need a symmetrical (balanced) power to find risk and protective minor alleles. One could argue that the direction is not important as long as the union of identified associations gets larger. However, subsequent interpretative analysis may be affected by this asymmetry [28–31]. In addition, rare variants that cause deleterious effects on a gene may have different clinical implication than rare variants that add a protective function to a gene [32]. Assessing how much this asymmetry may affect downstream analysis is beyond the scope of this study, but will certainly be an interesting topic for investigation in future studies.

In sum, in this study, we identified the power asymmetry problem of the current meta-analysis framework for overlapping controls and developed the solution, PASTRY. We believe that PASTRY will be the method of choice for meta-analyzing genomic studies that share controls, as it can provide balanced power for risk and protective minor alleles.

### Supplementary Information

**Additional file 1 Table S1.** 468 significant loci from the UK biobank Diabetes Mellitus GWAS results. **Table S2.** 8 significant loci related to the three autoimmune diseases (CD, RA, T1D) and the result of *p*-values of PASTRY method, LS method, and splitting approach for cross-disease meta-analysis of CD, RA, and T1D from the WTCCC data. **Table S3.** Power difference of PASTRY and LS at various MAF, RRs, the number of studies. **Fig. S1. **False positive rates of LS, PASTRY and splitting. **Fig. S2. **Quantile-Quantile (QQ) plots for the GWAS meta-analysis of three different models.

## Data Availability

The model proposed in this study are available in the GitHub repository: https://github.com/hanlab-SNU/PASTRY. To access WTCCC and UK Biobank genotype data, you need to apply through their respective websites. You can check the details at https://www.wtccc.org.uk/info/access_to_data_samples.html and https://www.ukbiobank.ac.uk/enable-your-research/apply-for-access. License: PASTRY is under the MIT license.

## Appendix: Detailed derivation of PASTRY

### Information matrix

Let ${X}_{ki}$ and ${Y}_{ki}$ denote the genotype and phenotype of the ${i}^{th}$ individual in study $k$, respectively. The maximum likelihood estimation (MLE) is widely used for estimating the parameters of a logistic regression model. The likelihood for the logistic regression model of study $k$ is

13 $$L\left({\alpha }_{k},{\beta }_{k}\right)= \prod_{i}^{{n}_{k}}\frac{{e}^{{(\alpha }_{k}+{{\beta }_{k}X}_{ki}){Y}_{ki}}}{1+{e}^{{\alpha }_{k}+{{\upbeta }_{k}X}_{ki}}},$$

where ${\alpha }_{k}$ and ${\beta }_{k}$ denote the intercept and regression parameters of study $k$, respectively.

Typically, a log-likelihood function is used to simplify the derivative. The log-likelihood function is

14 $${l}_{k}^{ }\left({\alpha }_{k},{\beta }_{k}\right)=\sum_{i}^{{n}_{k}}{Y}_{ki}({\alpha }_{k}+{{\beta }_{k}X}_{ki})-\sum_{i}^{{n}_{k}}ln\left(1+{e}^{{\alpha }_{k}+{{\beta }_{k}X}_{ki}}\right),$$

The corresponding score function, which is the gradient of log-likelihood function, is as follows:

15 $${U}_{k}\left({\alpha }_{k},{\beta }_{k}\right)= {l}^{\prime}\left({\alpha }_{k},{\beta }_{k}\right)=\sum_{i}^{{n}_{k}}\left({Y}_{ki}-\frac{{e}^{{\alpha }_{k}+{{\beta }_{k}X}_{ki}}}{1+{e}^{{\alpha }_{k}+{{\beta }_{k}X}_{ki}}}\right){\widetilde{{\varvec{X}}}}_{ki},$$

where $${\widetilde{{\varvec{X}}}}_{ki}=\left[\begin{array}{c}1\\ {X}_{ki}\end{array}\right]$$.

Next, the information matrix ${I}_{k}$ is the variance–covariance matrix of the score function. According to the information matrix equality [33], ${I}_{k}$ can be defined as:

16 $${I}_{k}\left({\alpha }_{k},{\beta }_{k}\right)=E[- {l}^{{\prime}{\prime}}\left({\alpha }_{k},{\beta }_{k}\right)]=\sum_{i}^{{n}_{k}}\frac{{e}^{{\alpha }_{k}+{{\beta }_{k}X}_{ki}}}{{\left(1+{e}^{{\alpha }_{k}+{{\beta }_{k}X}_{ki}}\right)}^{2}}{\widetilde{{\varvec{X}}}}_{ki}{\widetilde{{\varvec{X}}}}_{ki}^{T},$$

where $${\widetilde{{\varvec{X}}}}_{ki}{\widetilde{{\varvec{X}}}}_{ki}^{T}=\left[\begin{array}{cc}1& {X}_{ki}\\ {X}_{ki}& {X}_{ki}^{2}\end{array}\right]$$.

On the other side, we can get the odds function (from Eq. (1)) by inversing the standard logistic function and exponentiating both sides:

$$logit(p_{i})=\mathit{log}\left(\frac{p_{i}}{1-p_{i}}\right)={\alpha}_{k} +{\beta }_{k}{X}_{ki}+\epsilon_{i},$$

17 $$\frac{p_{i}}{1 - p_{i}} = { }e^{{\alpha_{k} + \beta_{k} X_{ki} }+\epsilon_{i}}.$$

We can modify this logit function to express it in terms of the estimate of ${e}^{\alpha }$:

$${e}^{{\hat{\alpha }_{k}}}\approx exp\left(log\left(\frac{\frac{{n}_{k+}}{{n}_{k+}+{n}_{k-}}}{\frac{{n}_{k-}}{{n}_{k+}+{n}_{k-}}}\right)-{\beta }_{k}\left(\frac{{MAF}_{k+}\times {n}_{k+}+{ M{AF}_{k-}\times n}_{k-} }{{n}_{k+}+{n}_{k-}}\right)\right)$$

18 $$= exp\left(log\left(\frac{{n}_{k+}}{{n}_{k-}}\right)-{\beta }_{k}\left(\frac{{MAF}_{+}\times {n}_{k+}+{ M{AF}_{-}\times n}_{k-} }{{n}_{k+}+{n}_{k-}}\right)\right),$$

where we replaced $p$ (probability of disease) with $\frac{{n}_{k+}}{{n}_{k+}+{n}_{k-}}$ and $1-p$ with $\frac{{n}_{k-}}{{n}_{k+}+{n}_{k-}}$ which are the expectations of $p$ and $1-p$ in the $k$ th study, respectively. ${X}_{k}$ is genotype score by definition, so we can reformulated it as $\frac{{MAF}_{+}\times {n}_{k+}+{M{AF}_{-}\times n}_{k-} }{{n}_{k+}+{n}_{k-}}$ which is the expectation of $X$ in the $k$ th study; ${n}_{k+}$ and ${n}_{k-}$ are the number of sample sizes of case and control in study $k$, and $MA{F}_{+}$ and $MA{F}_{-}$ denote the minor allele frequencies of case and control, respectively [15].

Since we don’t know the true beta (${\beta }_{k}$) of formula (18), we substitute the LS estimator ($\hat{{\beta}}_{LS}$) for ${\beta }_{k}$ in our method. Recall that from formula (8) above, LS estimator ${\beta }_{LS}$ is:

19 $$\hat{\beta}_{LS} = \frac{{e^{T} \Omega_{LS}^{ - 1} \hat{\beta }}}{{e^{T} \Omega_{LS}^{ - 1} e}}$$

Then, we can divide ${e}^{{\alpha }_{k}+{\beta }_{k}{X}_{ki}}$ (in Eq. (16)) into two cases:

If $${X}_{ki}=0,$$

20 $${e}^{{\alpha }_{k}+{\beta }_{k}{X}_{ki}}\approx{e}^{\hat{{\alpha }_{k}}},$$

and if $${X}_{ki}=1,$$

21 $${e}^{{\alpha }_{k}+{\beta }_{k}{X}_{ki}}\approx {e}^{\hat{{\alpha }_{k}}+\hat{{\beta}}_{LS}}.$$

Similarly, we can also calculate ${\widetilde{{\varvec{X}}}}_{ki}{\widetilde{{\varvec{X}}}}_{li}^{T}$ according to two ${X}_{ki}$:

If $${X}_{ki}=0,$$

22 $${\widetilde{{\varvec{X}}}}_{ki}{\widetilde{{\varvec{X}}}}_{ki}^{T}=\left[\begin{array}{cc}1& {X}_{ki}\\ {X}_{ki}& {X}_{ki}^{2}\end{array}\right]=\left[\begin{array}{cc}1& 0\\ 0& 0\end{array}\right],$$

and if $${X}_{ki}=1,$$

23 $${\widetilde{{\varvec{X}}}}_{ki}{\widetilde{{\varvec{X}}}}_{ki}^{T}=\left[\begin{array}{cc}1& {X}_{ki}\\ {X}_{ki}& {X}_{ki}^{2}\end{array}\right]=\left[\begin{array}{cc}1& 1\\ 1& 1\end{array}\right].$$

Now, we can write the information matrix of study $k$ as

24 $${I}_{PASTRY}\approx {n}_{k+}\left(1-{MAF}_{k+}\right)\frac{{e}^{\hat{{\alpha }_{k}}}}{{\left(1+{e}^{\hat{{\alpha }_{k}}}\right)}^{2}}\left[\begin{array}{cc}1& 0\\ 0& 0\end{array}\right]+{{n}_{k+}\times MAF}_{k+}\frac{{e}^{\hat{{\alpha }_{k}}+{\hat{\beta }_{LS}}}}{{\left(1+{e}^{\hat{{\alpha }_{k}}+{\hat{\beta}_{LS}}}\right)}^{2}}\left[\begin{array}{cc}1& 1\\ 1& 1\end{array}\right]+{n}_{k-}\left(1-{MAF}_{k-}\right)\frac{{e}^{\hat{{\alpha }_{k}}}}{{\left(1+{e}^{{\hat{\alpha }_{k}}}\right)}^{2}}\left[\begin{array}{cc}1& 0\\ 0& 0\end{array}\right]+{{n}_{k-}\times MAF}_{k-}\frac{{e}^{{\hat{\alpha }_{k}}+{\hat{\beta}}_{LS}}}{{\left(1+{e}^{\hat{{\alpha }_{k}}+ \hat{\beta}_{LS}}\right)}^{2}}\left[\begin{array}{cc}1& 1\\ 1& 1\end{array}\right] ,$$

where the general structure of information matrix, defined by formula (16), is decomposed into four parts, each representing specific conditions: (1) case group having non-risk allele (${X}_{ki}=0$ and ${Y}_{ki}=1$), (2) case group having risk allele (${X}_{ki}=1$ and ${Y}_{ki}=1$), (3) control group having risk allele (${X}_{ki}=1$ and ${Y}_{ki}=0$), and (4) control group having non-risk allele (${X}_{ki}=0$ and ${Y}_{ki}=0$). Next, each component in formula (16) is substituted with the corresponding formulas, namely formula (18), (20), (21), (22), and (23), which gives us the matrix.

### Covariance matrix

Let ${{\varvec{\theta}}}_{k}= ({\alpha }_{k}, {\beta }_{k})$ be the set of parameters for the logistic regression model in the study $k$. Then, according to the maximum likelihood theory, the MLE of ${{\varvec{\theta}}}_{k}$ follows:

25 $${\widehat{{\varvec{\theta}}}}_{{\varvec{k}}}\sim N\left({{\varvec{\theta}}}_{k},{I}_{k}^{-1}\left({{\varvec{\theta}}}_{{\varvec{k}}}\right)\right)$$

and the covariance between the parameters of two studies is known to be [15]:

26 $$Cov\left({\widehat{{\varvec{\theta}}}}_{k},{\widehat{{\varvec{\theta}}}}_{l}\right)\approx {{I}_{k}}^{-1}\left({{\varvec{\theta}}}_{k}\right) Cov\left({U}_{k}\left({{\varvec{\theta}}}_{k}\right),{U}_{l}\left({{\varvec{\theta}}}_{l}\right)\right){{I}_{l}}^{-1}\left({{\varvec{\theta}}}_{l}\right).$$

By the definition of covariance, the covariance between score functions of study $k$ and $l$:

$$Cov\left({U}_{k}\left({{\varvec{\theta}}}_{k}\right),{U}_{l}\left({{\varvec{\theta}}}_{l}\right)\right)$$

$$={\text{E}}\left[\left({{\text{U}}}_{k}\left({{\varvec{\theta}}}_{k}\right)-{\text{E}}\left[{{\text{U}}}_{k}\left({{\varvec{\theta}}}_{k}\right)\right]\right)\left({[{\text{U}}}_{l}\left({{\varvec{\theta}}}_{l}\right)-{\text{E}}\left[{{\text{U}}}_{l}\left({{\varvec{\theta}}}_{l}\right)\right]\right)\right]$$

$$={\text{E}}\left[\left({{\text{U}}}_{k}\left({{\varvec{\theta}}}_{k}\right)\right)\left({{\text{U}}}_{l}\left({{\varvec{\theta}}}_{l}\right)\right)\right]$$

27 $$\approx \sum_{i}^{{n}_{kl}}\left({Y}_{ki}-\frac{{e}^{{\alpha }_{k}+{{\beta }_{k}X}_{ki}}}{1+{e}^{{\alpha }_{k}+{{\beta }_{k}X}_{ki}}}\right)\left({Y}_{li}-\frac{{e}^{{\alpha }_{l}+{{\beta }_{l}X}_{li}}}{1+{e}^{{\alpha }_{l}+{{\beta }_{l}X}_{li}}}\right){\widetilde{{\varvec{X}}}}_{ki}{\widetilde{{\varvec{X}}}}_{li}^{T} ,$$

where ${n}_{kl}$ denotes the number of overlapping samples between two studies ($k$ and $l$). Here, we can decompose overlapping samples to cases and controls:

$$Cov\left({U}_{k}\left({{\varvec{\theta}}}_{k}\right),{U}_{l}\left({{\varvec{\theta}}}_{l}\right)\right)\approx \left\{\sum_{i}^{{n}_{kl+}}\left(\frac{1}{1+{e}^{{\alpha }_{k}+{{\beta }_{k}X}_{ki}}}\right)\left(\frac{1}{1+{e}^{{\alpha }_{l}+{{\beta }_{l}X}_{li}}}\right)+\sum_{i}^{{n}_{kl-}}\left(-\frac{{e}^{{\alpha }_{k}+{{\beta }_{k}X}_{ki}}}{1+{e}^{{\alpha }_{k}+{{\beta }_{k}X}_{ki}}}\right)\left(-\frac{{e}^{{\alpha }_{l}+{{\beta }_{l}X}_{li}}}{1+{e}^{{\alpha }_{l}+{{\beta }_{l}X}_{li}}}\right)\right\}{\widetilde{{\varvec{X}}}}_{ki}{\widetilde{{\varvec{X}}}}_{li}^{{\varvec{T}}},$$

28 $$\approx \left\{\sum_{i}^{{n}_{kl-}}\left(-\frac{{e}^{{\alpha }_{k}+{{\beta }_{k}X}_{ki}}}{1+{e}^{{\alpha }_{k}+{{\beta }_{k}X}_{ki}}}\right)\left(-\frac{{e}^{{\alpha }_{l}+{{\beta }_{l}X}_{li}}}{1+{e}^{{\alpha }_{l}+{{\beta }_{l}X}_{li}}}\right)\right\}{\widetilde{{\varvec{X}}}}_{ki}{\widetilde{{\varvec{X}}}}_{li}^{T},$$

where we can put ${n}_{kl+}$ term as zero because we only consider the shared controls in this study. The corresponding variance–covariance matrix of score functions between study $k$ and $l$ is therefore,

29 $$Cov_{PASTRY}\left({U}_{k}\left({\widehat{\varvec{\theta}}}_{k}\right),{U}_{l}\left({\widehat{\varvec{\theta}}}_{l}\right)\right)\approx {n}_{kl-}\left(1-{MAF}_{-}\right)\frac{{e}^{{\hat{\alpha}_{k}}+\hat{{\alpha}_{l}}}}{\left(1+{e}^{\hat{{\alpha }_{k}}}\right)\left(1+{e}^{\hat{{\alpha }_{l}}}\right)}\left[\begin{array}{cc}1& 0\\ 0& 0\end{array}\right]+{{n}_{kl-} MAF}_{-}\frac{{e}^{(\hat{{\alpha }}_{k}+\hat{{\beta }}_{LS})+{(\hat{\alpha}}_{l}+\hat{{\beta}}_{LS})}}{\left(1+{e}^{\hat{{\alpha }}_{k}+\hat{{\beta }}_{LS}}\right)\left(1+{e}^{{\hat{\alpha}}_{l}+{\hat{\beta }}_{LS}}\right)}\left[\begin{array}{cc}1& 1\\ 1& 1\end{array}\right],$$

where each component in formula (27), representing the general variance–covariance matrix of score function formula, is decomposed into two parts, excluding ${n}_{kl+}$ part. Subsequently, we replaced these components with the corresponding formulas, namely formulas (18), (20), (21), (22), and (23), which gives us the matrix. Lastly, we can get the variance–covariance of PASTRY between study $k$ and $l$ (denoted in formula (10)) using formula (26).
