# Supplementary material for: PASTRY: achieving balanced power for detecting risk and protective minor alleles in meta-analysis of association studies with overlapping subjects
Source: BMC Bioinformatics. 2024 Jan 12;25:24. doi: 10.1186/s12859-023-05627-z (PMC10790263; doi:10.1186/s12859-023-05627-z)
Supplement: Supplementary file 1 — Additional file 1 Table S1. 468 significant loci from the UK biobank Diabetes Mellitus GWAS results. Table S2. 8 significant loci related to the three autoimmune diseases (CD, RA, T1D) and the result of p-values of PASTRY method, LS method, and splitting approach for cross-disease meta-analysis of CD, RA, and T1D from the WTCCC data. Table S3. Power difference of PASTRY and LS at various MAF, RRs, the number of studies. Fig. S1. False positive rates of LS, PASTRY and splitting. Fig. S2. Quantile-Quantile (QQ) plots for the GWAS meta-analysis of three different models. [file 12859_2023_5627_MOESM1_ESM.zip › BMC_Bioinformatics_PASTRY_Supplementary_figure.pdf]

Supplementary Figure of " PASTRY: Achieving balanced power for detecting risk and protective minor alleles in meta-analysis of association studies with overlapping subjects."

Emma E. Kim[1†], Chloe Soohyun Jang[2†], Hakin Kim[3] and Buhm Han[1,3*]

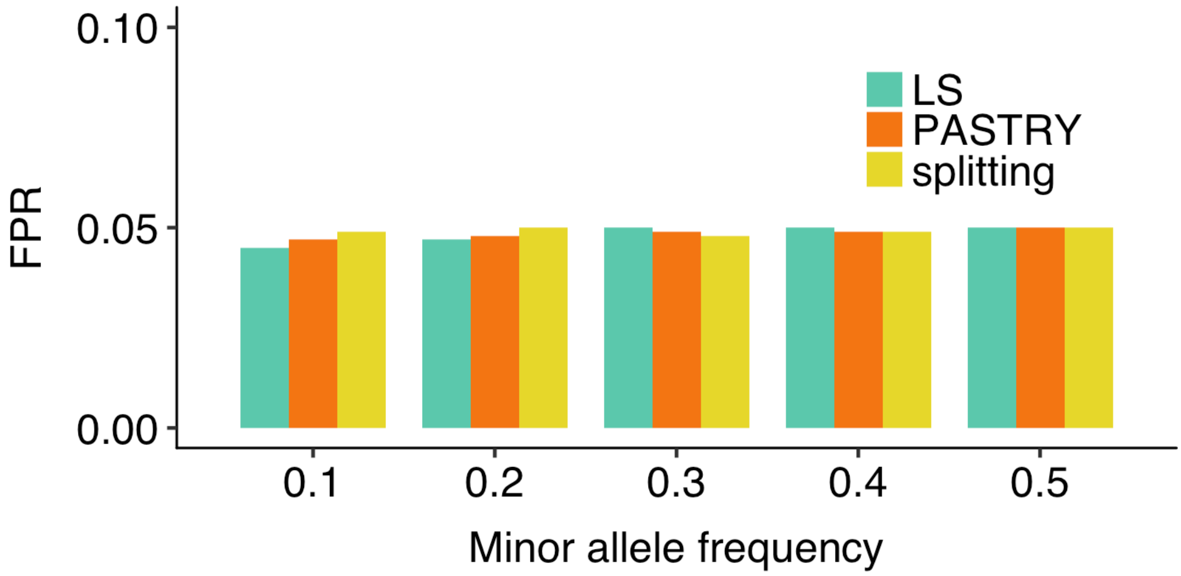

Supplementary Figure S1. False positive rates of LS, PASTRY and splitting. The X-axis indicates the minor allele frequency (MAF) and the Y-axis indicates the false-positive rate given the significance threshold α = 0.05.

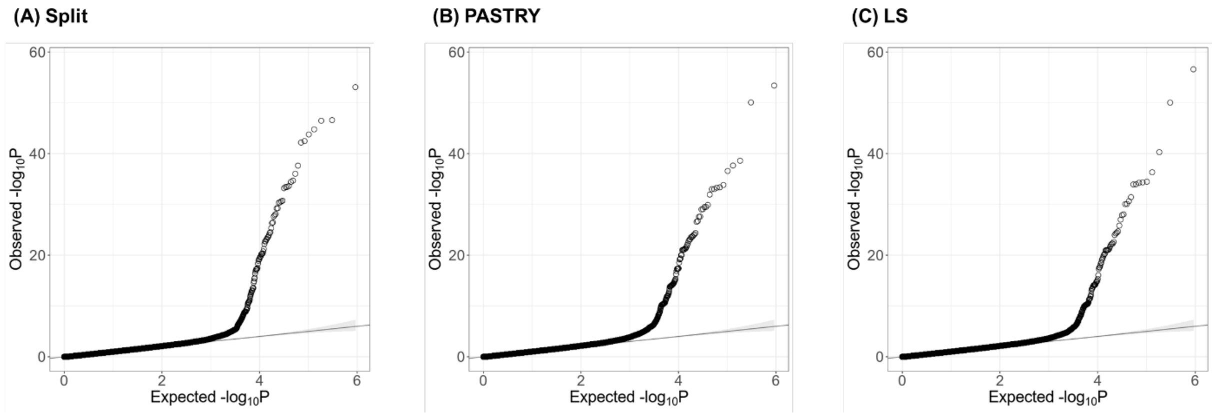

Supplementary Figure S2. Quantile-Quantile(Q-Q) plots for the GWAS meta-analysis of three different models for all SNPs. The X-axis indicates the expected -log10-transformed P-values and the Y-axis indicates the observed -log10-transformed P-values.
